# Influential Factors of Burnout among Village Doctors in China: A Cross-Sectional Study

**DOI:** 10.3390/ijerph18042013

**Published:** 2021-02-19

**Authors:** Xinyi Zhao, Shu Liu, Yifan Chen, Quan Zhang, Yue Wang

**Affiliations:** 1 Department of Medical Ethics and Law, School of Health Humanities, Peking University, Beijing 100191, China; zhaoxinyi@hsc.pku.edu.cn; 2 Department of Language and Culture in Medicine, School of Health Humanities, Peking University, Beijing 100191, China; ls13161511366@pku.edu.cn (S.L.); chen_yf@pku.edu.cn (Y.C.); 3 National School of Development, Peking University, Beijing 100871, China; zhangquan1995@pku.edu.cn

**Keywords:** burnout, village doctors, rural healthcare system, influential factors

## Abstract

(1) Background: The heavy workload and understaffed personnel of village doctors is a challenge to the rural healthcare system in China. Previous studies have documented the predictors of doctors’ burnout; however, little attention has been paid to village doctors. This study aims to investigate the prevalence and influential factors of burnout among village doctors. (2) Methods: Data was collected by a self-administered questionnaire from 1248 village doctors who had worked at rural clinics for more than a year. Burnout was measured using the Maslach Burnout Inventory-Human Services Survey (MBI-HSS) with three dimensions—emotional exhaustion (EE), depersonalization (DP), and reduced personal accomplishment (PA). A logistic regression model was applied to estimate the influential factors of burnout. (3) Results: The prevalence of overall burnout was 23.6%. Being male (OR = 0.58, 95%CI: 0.41–0.82), poor health status (OR = 0.80, 95%CI: 0.67–0.94), low income (OR = 0.62, 95%CI: 0.40–0.95), and a poor doctor–patient relationship (OR = 0.57, 95%CI: 0.48–0.67) were significantly related to burnout. Conclusion: Burnout is prevalent among Chinese village doctors. Policies such as increasing village doctors’ income and investing more resources in rural healthcare system should be carried out to mitigate and prevent burnout.

## 1. Introduction

Burnout is a psychological syndrome experienced when people face an accumulation of emotional exhaustion and cynicism due to work-related stress [1]. It is characterized by three dimensions: (1) emotional exhaustion (EE)—an increased feeling of fatigue due to depleted emotional resources; (2) depersonalization (DP)—negative and cynical attitudes towards clients and partners; and (3) reduced personal accomplishment (PA)—a tendency to undervalue work-related competency and achievement [2]. Burnout in physicians results in a range of negative consequences: mental health problems including anxiety, depression, and even suicidal thoughts [3,4,5,6], lower job satisfaction, a higher turnover intention [7,8,9,10], increased medical errors and reduced quality of healthcare [8,9,11,12]. Village doctors play an essential role in providing primary healthcare for a large number of rural residents in China, but they are confronted by challenges of understaffing and an overload of patients. Identifying the prevalence and predictors of village doctors’ burnout is of great importance as it provides implications for prevention and intervention.

Village doctors are indispensable health personnel in China, a country with a rural population of 551.6 million in 2019 [13]. They are responsible for the basic public health and medical services, such as diagnosis and treatment towards common diseases, in the village health institutions. Village doctors in China were known as “barefoot doctors” when they began practicing in the mid-1950s [14]. Barefoot doctors then were the executors of the Rural Cooperative Medical System (RCMS), providing primary medical services to rural residents. The World Health Organization regarded the barefoot doctor system as a successful example of healthcare provision in developing regions with inadequate resources [15]. Due to the limitation of education and healthcare resources at that time, barefoot doctors did not receive higher education, and even many of them were peasants with basic medical trainings. They had a training programme of at least three months duration and refresher courses thereafter to increase their knowledge and ability [16]. In 1985, the Ministry of Health stopped using the term barefoot doctors; those who passed a government assessment qualified as “village doctors” [14,15]. However, an unsuccessful healthcare system reform in the 1980s led to the collapse of the RCMS, the loss of institutional and financial support for the village doctors, and a lack of healthcare services for rural residents [17]. In 2003, the Government initiated the New Rural Cooperative Medical System (NRCMS) and announced “Deepening the Reform of the Medical and Health Care System” in 2009. Nonetheless, reform in the rural healthcare system was impeded by a shortage of personnel. According to the China Health Statistic Yearbook 2019, the number of village doctors decreased in the past decade. In 2011, there were 1.1 million village doctors and medical orderlies (i.e., staff working in a village clinic but not certified as village doctors) in rural areas, accounting for 13.1% of all health workers in China. However, in 2019, the number of village doctors and medical orderlies in rural areas reduced to 0.9 million, and the proportion to the total number of health workers fell to 7.4% [18]. In addition, the education requirement for village doctors is relatively low according to the “Regulation on the Administration of Village Doctors”. They are only required to have diploma of (above) technical secondary school in medicine; or have been worked in village health institution for (more than) 20 years consecutively; or have accepted the training programme set forth by the local administrative department for health. While practicing medical doctors should have bachelor’s degree or higher and assistant practicing medical doctors should at least receive education from three-year college in medicine, based on the “Law on Practicing Doctors of the People’s Republic of China”. Hence, a very small number of village doctors have received college education and obtained the qualifications of (assistant) practicing medical doctors.

Burnout has been described as a global phenomenon among health professionals [19]. Previous research has probed into the prevalence and predictors of burnout among different groups of health professionals, including surgeons [20], dentists [21], neurologists [22], and general practitioners [23], etc. Although health workers in primary healthcare facilities provide relatively less complicated services than those of surgeons or neurologists, they may nonetheless report burnout due to limited resources and a poor working environment [24,25]. The risk factors in doctors’ burnout have been well-documented in prior research [20,21,22,25,26,27,28] including (1) socio-demographic characteristics such as age, sex, and education level; and (2) work-related factors such as working hours and doctor–patient relationship. Existing literature on burnout in Chinese physicians has mainly focused on those in urban areas or higher-level hospitals, with studies on physicians or general practitioners in Chinese urban hospitals indicating a high level of burnout [23,29]. Considering the working environment, duties, as well as the education levels are quite different between urban and rural doctors, it is important to extend understanding on the rural group. However, relevant studies are limited. Some have assessed the development and status quo of village doctors/barefoot doctors [15,16,17,30], and some empirical studies have found that village doctors reported a low level of job satisfaction [31], and that their income was reduced after the healthcare system reform of 2009 [32]. A study on health workers in county-level facilities in Shandong province revealed an association between contextual factors (such as institutional support) and burnout [29]. Another study focused on primary healthcare workers at township hospitals in six provinces and found factors such as long working hours, young age, and a college education were related to burnout [25]. Hence, there is a lack of research exploring the burnout of doctors in rural areas in China, especially those working at village clinics. In addition, research on rural doctors in Western regions is also limited. A survey in British Columbia demonstrated that among family physicians in rural communities, 54% suffered from high EE, 30% suffered from high DP, and 18% had low PA [33]. A study on rural physicians in emergency medicine in Southwestern Ontario showed 85.7% of the participants reported burnout [34]. Although rural doctors in both eastern and western world may face excessive workload, deeply influenced by Confucian culture, Chinese people are expected to fulfill their duty without complaining even when the workload is overwhelming [9]. It would be interesting to investigate the burnout of rural doctors under Chinese culture. Therefore, in the present study, we focus on village doctors and investigate the prevalence of burnout and the multiple influential factors that contribute to it among village doctors in China.

## 2. Materials and Methods

### 2.1. Study Participants and Data Collection

The study collected data by convenience and purposive sampling. The participants were village doctors who had worked at rural clinics for at least a year. Taking advantage of a training programme which was held in Beijing and open to village doctors throughout the country, we approached 16 classes involving 1280 village doctors. Electronic questionnaires were delivered through an online survey software “Wen Juan Xing” (Ranxing Information Technology Limited, Changsha, China), and the participants were asked to fill in the self-administered questionnaire on their smart phones. The survey was anonymous. We received 1248 eligible questionnaires, with a response rate of 97.5%. All the participants provided informed consent. The study protocol was approved by the Human Research Ethics Committee at the authors’ institution.

### 2.2. Measures

Burnout was assessed by the Chinese version of MBI-Human Services Survey (MBI-HSS) [35] which has been widely used in research on Chinese health professionals (e.g., [23,29,36]). The MBI-HSS has 22 items with a 6-point Likert scale ranging from 0 (never) to 6 (every day). It consists of three subscales, namely, emotional exhaustion (EE; nine items), depersonalization (DP; five items), and personal accomplishment (PA; eight items). The potential scores range from 0 to 54 for EE, from 0 to 30 for DP, and from 0 to 48 for PA. Higher scores of EE and DP, and lower scores of PA, indicate higher risks of burnout. Overall burnout was determined as a high EE score along with a high DP score or a low PA score. This approach was recommended by Maslach (in a personal communication in 2008) [37] and other scholars [38], and also applied in previous research [39]. Base on prior research [23,29,36], the cut-offs for high EE, high DP, and low PA in the definition of overall burnout were: EE ≥ 27, DP ≥ 13, and PA ≤ 31, respectively. We noticed that the prevalence of burnout even varied in studies using the same tool (MBI-HSS) because of heterogeneity in their definitions on overall burnout [19]. Some used a more stringent criterion that defined burnout as high EE, high DP, and low PA (e.g., [23]); while some applied a looser criterion that defined burnout as high scores of either EE or DP (e.g., [34,36]). In order to facilitate the comparison with previous studies, we also conducted supplementary calculations on burnout rates according to the two approaches. In the present study, Cronbach’s alpha coefficients for the MBI-HSS and its three subscales—EE, DP, and PA, were 0.860, 0.928, 0.841, and 0.784, respectively. 

Other variables related to the participants’ socio-demographic and work conditions were included. The socio-demographic variables were age (a continuous variable); sex (male or female); education (high school or below, associate degree, bachelor or above); pension status (covered pension scheme or not); and self-rated health (a 5-point scale with responses ranging from 1 = very poor to 5 = very good). Job-related variables in the analyses were after-tax monthly income (yuan); healthcare work experience (years); average work time per day (hours); vacation leave (vacation leave taken last year or not); and the perception of doctor–patient relationship experienced (a 5-point scale with responses ranging from 1 = very poor to 5 = very good).

### 2.3. Statistical Analysis

Descriptive analyses were conducted to understand the characteristics and prevalence of burnout in the participants. A logistic regression model was applied to estimate the influential factors of village doctors’ burnout. Dependent variables—burnout was treated as a dichotomous variable. Potential predictors such as age, sex, educational attainment, marital status, self-rated health, pension scheme, monthly income, working hours per day, years of working, vacation leave, and doctor–patient relationship were included in the regression models. The statistical analyses were performed with SPSS version 26 (IBM Corp, Armonk, NY, USA).

## 3. Results

### 3.1. Respondents’ Characteristics 

Table 1 shows the demographic and job characteristics of the respondents. The average age was 46.12 (SD = 7.75) and more than half (56.09%) were between 40 and 49 years old. The respondents were predominantly male (71.07%) and married (95.43). Among the participants, 67.31% had a high school education or below, 27% had obtained an associated degree (i.e., finished the three-year college education), while only 5.69% had a bachelor’s degree or higher. The majority rated their health status as average or above; only 9.21% and 3.29% reported poor and very poor health, respectively. About one-fifth (18.27%) of the respondents were not covered by any pension scheme. Among the respondents, experience in the medical profession was 24.40 years (SD = 8.99) on average, and mean working hours per day were 13.56 (SD = 4.87). Most (93.99%) of the respondents had not had any vacation leave in the past year. The average income per month was 2,878.92 (SD = 11,859.24) yuan. Nearly half (45.75%) of the participants rated the doctor–patient relationship in their work environment as average, followed by those rating it as good (31.73%), very good (12.74%), bad (6.89%), and very bad (2.88%).

### 3.2. Prevalence of Burnout

As the most recent MBI manual (fourth edition) has suggested not to accumulate the scores of three dimensions to form a single burnout score [40], we analyzed the mean scores of each dimension. As shown in Table 2, the mean scores of EE, DP, and PA were 29.61 (SD = 16.19), 4.84 (SD = 6.74), and 36.03 (SD = 10.16), respectively. Of the participants, 23.6% reported high EE scores (≥27) together with high DP scores (≥13) or low PA scores (≤31), indicating that the prevalence of overall burnout among village doctors was 23.6% according to the definition by existing literature [37,38]. If we calculated overall burnout by the criterion of high EE, high DP, and low PA, or the criterion of high EE or high DP, the rate of overall burnout was 4.4% and 56.3%, respectively.

### 3.3. Factor Associated with Burnout

Table 3 shows factors related to the participants’ burnout. Male village doctors (*p* < 0.01, OR = 0.58, 95%CI: 0.41–0.82) were more likely to have burnout. Poorer health status was significantly related to burnout (*p* < 0.01, OR = 0.80, 95%CI: 0.67–0.94). In terms of work-related factors, participants with lower income had a higher risk of burnout (*p* < 0.05, OR = 0.62, 95%CI: 0.40–0.95). Those who experienced a poorer doctor-patient relationship were more likely to report burnout (*p* < 0.001, OR = 0.57, 95%CI: 0.48–0.67). 

## 4. Discussion

By using self-collected data from 1248 village doctors in China, this study shed light on the prevalence of burnout and related factors among village doctors in China. Our results revealed a high prevalence of burnout among village doctors, and that burnout could be explained by the following variables: sex, self-rated health, income, and the doctor–patient relationship.

The prevalence of burnout among village doctors in our study was slightly lower than the findings by O’Kelly and colleagues [39] on urologists in the UK (28.9% also measured by high EE plus high DP or low PA), as well as Li and colleagues [36] on Chinese anesthesiologists (69% measured by either high EE or high DP). It might because work responsibilities for specialist physicians in urban hospitals seem more demanding than those for village doctors. However, if compared with primary caregivers in urban areas, the prevalence of burnout among village doctors was higher. Gan and colleagues [23] showed that 2.46% of general practitioners in urban areas in Hubei province of China reported burnout (measured by high EE, high DP, and low PA), which was lower than the figure in our study. It might be attributed to a harder living and working environment and a much lower income than primary health providers in urban regions. According to the China Health Statistic Yearbook 2019, the number of health professionals per 10,000 people is 18 and 40 in rural and urban areas, respectively [18]. An overload of patients, combined with insufficient resources in the rural healthcare system, has increased the workload among village doctors over that of their urban counterparts. The demands of their job exceeded their capabilities, resulting in symptoms of burnout. Moreover, compared with a study on rural emergency medicine physicians in Southwestern Ontario reporting burnout rate as 85.7% (defined by high EE or high DP), the figure in our study was smaller. One possible reason is work intensity of emergency medicine may be higher than that of village clinics. Another reason might be that influenced by Confucian culture, village doctors in China are prone to absorbing the negative feelings internally when facing exhausting tasks, while in similar situations some western rural doctors choose to protest and withdraw their services [9]. 

Sociodemographic factors including sex and self-rated health were found to be related to overall burnout among village doctors. The female participants were less likely to report burnout than the male counterparts. Previous studies did not reach a consensus on whether females or males were more likely to report burnout [6]. For instance, research on physicians in Shanghai [26] and surgeons in US [41] showed that females had higher risks of burnout, while studies on Dutch dentists [42] and European family doctors [43] indicated the link between male sex and burnout. Actually, gender is confounded with many other factors, such as occupations and culture-specific social roles and expectations [44]. The finding in our study could be explained by the following reasons: first, the cases in village clinics may be less complicated than those in urban hospitals; second, working as a doctor—a respected occupation, may boost the self-actualization and social status of females in rural areas, thus contributing to high PA; and third, females may be better at handling interpersonal relationships, so they may experience better doctor–patient relationship and have low DP. As expected, poorer self-rated health was significantly associated with overall burnout among village doctors. This could be explained by the fact that better health enables doctors to withstand stress and work efficiently with lower levels of burnout. The association between age and burnout was not significant, in contrast with previous research identifying a correlation between younger age and burnout [25,26]. One plausible explanation could be that in developed areas or higher-level hospitals, younger doctors are usually inexperienced in confronting complicated cases, leading to burnout. Village doctors in the present study mostly dealt with common cases such as fever and chronic diseases among villagers, so their age did not pose a significant impact on daily performance and burnout. Although education level was not found to be related with burnout in the present study, we should notice the fact that the low education level among the participants—more than two-thirds (67.31%) of them did not receive college education. As the serious urban–rural disparity in China, majority of graduates from medical colleges do not prefer to work in village-level medical institutions. Therefore, the educational requirement for village doctors is much lower than that for practicing medical doctors or assistant medical doctors who usually work in urban hospitals. As the low education level of health personnel might impact the quality of healthcare, and even cause medical errors, the government should take more effective measures to attract medical students to work in rural areas and improve the capabilities of current village doctors. 

Job-related factors are equally important. Our results showed a significant negative association of income with burnout scores. Similarly, previous studies reported that income was positively related to job satisfaction [31] and negatively related to burnout among physicians [22]. According to the theory of effort-reward imbalance [45], an imbalance between cost and gain generates emotional distress among health workers [46]. Tremendous effort in the work of village doctors but low-income rewards would increase burnout. The association between working hours per day and overall burnout was not significant in the present study (*p* = 0.07). This finding was inconsistent with previous studies on doctors in high-level health institutions. For example, in a focus on physicians from hospitals in Shanghai, Wang and colleagues reported higher risks of burnout linked to longer working hours [26]. Xu and colleagues also found that doctors in township hospitals were more likely to have burnout if they worked for longer hours per week [25]. A possible explanation is that village doctors’ work tasks might be relatively easy to handle. Therefore, although the participants worked up to 13.56 h per day, their tasks did not directly increase work intensity which may cause chronic stress [47]. However, it is obvious that the 13.56 working hours per day exceeds the legal working hours in China (i.e., 8 h). This might because limited number of village doctors provided primary healthcare for a large number of rural residents. Moreover, as village doctors live in the same community with rural residents, usually they would not refuse a visit from a patient at nonworking time. Only 6.89 % and 2.88% of our participants perceived the doctor–patient relationship in their work environment as poor or very poor, respectively, indicating that doctor–patient relationship in rural settings might be relatively more harmonious than that in urban hospitals where more than 40% of participants reported dissatisfaction in their relationship with patients [12]. This could be due to a high degree of acquaintance in China’s countryside [48]. Village doctors live in the same village as their patients and are likely to have more communication with and understanding of the patients, establishing a more harmonious doctor–patient relationship. Although there were relatively good doctor–patient relationships in the participants’ work settings, a poor doctor–patient relationship was still a predictor of burnout, coinciding with findings of previous studies [12,27]. The relationship between physicians and patients involves a great deal of emotional demand [49]. A poor doctor–patient relationship is usually related to disputes, conflicts, or violence [50], which could heighten tension in the doctors’ working environment and increase incidences of burnout. 

Our findings could provide some implications on preventing and mitigating the burnout of village doctors. First, individual-level solutions such as mindfulness-based stress reduction may be effective [6]. Such interventions could be provided to village doctors, especially for the males and those with poor health status. Second, although burnout is a subjective individual experience, it is also rooted in society [51]. The government should take various measures to relieve village doctors’ burnout. For instance, it is necessary to allocate more financial support to rural healthcare system, so as to increase village doctors’ income and improve their work environment. Furthermore, the government should make efforts to increase the quantity and quality of rural health personnel. It is suggested to train medical students who are targeted to work in rural areas free of charge; or encourage retired doctors from township hospital to work in village clinics. Additionally, the “Law of the People’s Republic of China on the Promotion of Basic Medical and Health Care”, which has come into effect since 2020 stipulates that a practicing physician to be promoted to a deputy senior technical title shall have at least one year experience in medical institutions at or below the county level. Thus, the authorities could encourage urban medical doctors to work in village clinics for a period of one year, which would help reduce village doctors’ burden. Moreover, village doctors’ workload would even increase during the COVID-19 epidemic. They have been playing an essential role in epidemic prevention and control at grass-roots level by providing door-to-door services, including health education, measuring body temperature, disinfecting, psychological counseling, etc. Therefore, more support should be provided to village doctors at this time. 

This study has several limitations. First, data was collected using convenience sampling, not representative of the experience of all village doctors in China, a country with diverse regions. Cautious consideration should be taken when making generalizations from our results. Second, the cross-sectional study design made it difficult to identify causal relationships. Further study with a longitudinal design is needed. Third, data was collected by self-report of the respondents, which could cause recall bias. These limitations should be considered in future studies.

## 5. Conclusions

Despite its limitations, this study has added valuable information to the existing literature on health professionals by investigating the prevalence of burnout and its influential factors among Chinese village doctors. The prevalence of burnout among village doctors was about 23.6%. Burnout was associated with male gender, poor health, low income, and a poor doctor–patient relationship. Our findings highlight the necessity of government to improve support and resources for village doctors and rural healthcare systems, thereby preventing and mitigating burnout.

## Figures and Tables

**Table 1:** Respondents’ characteristics.

Variables	*n*	%
Age group		
<40	195	15.63
40–49	700	56.09
≥50	353	28.29
Sex		
male	887	71.07
female	361	28.93
Education		
≤high school	840	67.31
associate degree	337	27
≥bachelor’s degree	71	5.69
Marital status		
other than married	57	4.57
married	1191	95.43
Self-rated health		
very poor	41	3.29
poor	115	9.21
average	671	53.77
good	282	22.6
very good	139	11.14
Pension		
no	228	18.27
yes	1020	81.73
Vacation leave		
no	1173	93.99
yes	75	6.01
Doctor–patient relationship		
very bad	36	2.88
bad	86	6.89
average	571	45.75
good	396	31.73
very good	159	12.74
	Mean	SD
Years of experiences	24.4	8.99
Work hours per day	13.56	4.87
Monthly income	2878.92	11,859.24

**Table 2:** Scores of dimensions of Maslach Burnout Inventory-Human Services Survey (MBI-HSS) among village doctors.

Dimension	Mean	SD
EE	29.61	16.19
DP	4.84	6.74
PA	36.03	10.16

Note: emotional exhaustion (EE), depersonalization (DP) and personal accomplishment (PA).

**Table 3:** Logistic regression analysis of influential factors with burnout of village doctors.

Variable	Coefficient	SE	*p* Value	OR	(95%CI)
Age (years)	−0.02	0.02	0.420	0.98	(0.94, 1.03)
Sex (female)	−0.55	0.18	0.002	0.58	(0.41, 0.82)
Marriage (married)	0.34	0.37	0.360	1.40	(0.68, 2.90)
Education level					
≥bachelor or above	−0.14	0.33	0.682	0.87	(0.46, 1.67)
associate degree	0.06	0.16	0.726	1.06	(0.77, 1.46)
≤high school (ref)					
Self-rated health (range 1–5)	−0.23	0.08	0.007	0.80	(0.67, 0.94)
Pension (yes)	−0.23	0.17	0.195	0.80	(0.57, 1.12)
Monthly income (yuan) ^a^	−0.49	0.22	0.030	0.62	(0.40, 0.95)
Years of working	0.00	0.02	0.961	1.00	(0.96, 1.04)
Working hours per day	0.03	0.01	0.070	1.03	(1.00, 1.06)
Vocation leave (yes)	−0.01	0.34	0.986	0.99	(0.51, 1.95)
Doctor–patient relationship (range 1–5)	−0.57	0.09	0.000	0.57	(0.48, 0.67)

Note: ^a^ Monthly income was log-transformed into the linear regression analysis.

## Data Availability

The data presented in this study are available on request from the corresponding author.

## References

[1] Maslach C. (2003). Job burnout: New directions in research and intervention. Curr. Dir. Psychol. Sci..

[2] Maslach C., Jackson S.E., Leiter M.P. (1996). Maslach Burnout Inventory.

[3] Wright A.A., Katz I.T. (2018). Beyond burnout—Redesigning care to restore meaning and sanity for physicians. N. Engl. J. Med..

[4] Rogers M.E., Creed P.A., Searle J. (2014). Emotional labour, training stress, burnout, and depressive symptoms in junior doctors. J. Vocat. Educ. Train..

[5] Sonneck G., Wagner R. (1996). Suicide and burnout of physicians. Omega J. Death Dying.

[6] West C.P., Dyrbye L.N., Shanafelt T.D. (2018). Physician burnout: Contributors, consequences and solutions. J. Intern. Med..

[7] Zhang Y., Feng X. (2011). The relationship between job satisfaction, burnout, and turnover intention among physicians from urban state-owned medical institutions in Hubei, China: A cross-sectional study. BMC Health Serv. Res..

[8] Tawfik D.S., Profit J., Morgenthaler T.I., Satele D.V., Sinsky C.A., Dyrbye L.N., Tutty M.A., West C.P., Shanafelt T.D. (2018). Physician burnout, well-being, and work unit safety grades in relationship to reported medical errors. Mayo Clin. Proc..

[9] Lo D., Wu F., Chan M., Chu R., Li D. (2018). A systematic review of burnout among doctors in China: A cultural perspective. Asia Pac. Fam. Med..

[10] Wang H., Jin Y., Wang D., Zhao S., Sang X., Yuan B. (2020). Job satisfaction, burnout, and turnover intention among primary care providers in rural China: Results from structural equation modeling. BMC Fam. Pract..

[11] Salyers M.P., Bonfils K.A., Luther L., Firmin R.L., White D.A., Adams E.L., Rollins A.L. (2017). The relationship between professional burnout and quality and safety in healthcare: A meta-analysis. J. Gen. Intern. Med..

[12] Wu H., Liu L., Wang Y., Gao F., Zhao X., Wang L. (2013). Factors associated with burnout among Chinese hospital doctors: A cross-sectional study. BMC Public Health.

[13] National Data 2020. http://data.stats.gov.cn/easyquery.htm?cn=C01&zb=A0301&sj=2019.

[14] Cui W. (2008). China’s village doctors take great strides. Bull. World Health Organ..

[15] Zhang D., Unschuld P.U. (2008). China’s barefoot doctor: Past, present, and future. Lancet.

[16] Gong Y.L., Chao L.M. (1982). The role of barefoot doctors. Am. J. Public Health.

[17] Zhu N.S., Ling Z.H., Shen J., Lane J.M., Hu S.L. (1989). Factors associated with the decline of the Cooperative Medical System and barefoot doctors in rural China. Bull. World Health Organ..

[18] National Health Commission (2019). China Health Statistic Yearbook 2019.

[19] Rotenstein L.S., Torre M., Ramos M.A., Rosales R.C., Guille C., Sen S., Mata D.A. (2018). Prevalence of burnout among physicians: A systematic review. J. Am. Med. Assoc..

[20] Dimou F.M., Eckelbarger D., Riall T.S. (2016). Surgeon burnout: A systematic review. J. Am. Coll. Surg..

[21] Singh P., Aulak D.S., Mangat S.S., Aulak M.S. (2016). Systematic review: Factors contributing to burnout in dentistry. Occup. Med..

[22] Zhou X., Pu J., Zhong X., Zhu D., Yin D., Yang L., Zhang Y., Fu Y., Wang H., Xie P. (2017). Burnout, psychological morbidity, job stress, and job satisfaction in Chinese neurologists. Neurology.

[23] Gan Y., Jiang H., Li L., Yang Y., Wang C., Liu J., Yang T., Opoku S., Hu S., Xu H. (2019). Prevalence of burnout and associated factors among general practitioners in Hubei, China: A cross-sectional study. BMC Public Health.

[24] Arigoni F., Bovier P.A., Mermillod B., Waltz P., Sappino A.-P. (2009). Prevalence of burnout among Swiss cancer clinicians, paediatricians and general practitioners: Who are most at risk?. Support. Care Cancer.

[25] Xu W., Pan Z., Li Z., Lu S., Zhang L. (2020). Job burnout among primary healthcare workers in rural china: A multilevel analysis. Int. J. Environ. Res. Public Health.

[26] Wang Z., Xie Z., Dai J., Zhang L., Huang Y., Chen B. (2014). Physician burnout and its associated factors: A cross-sectional study in Shanghai. J. Occup. Health.

[27] Jesse M.T., Abouljoud M., Eshelman A. (2015). Determinants of burnout among transplant surgeons: A national survey in the United States. Am. J. Transplant..

[28] Cheng Y., Wang F., Zhang L., Zhang P., Ye B., Sun Y., Zhu X., Zhang N., Liang Y. (2019). Effects of organisational and patient factors on doctors’ burnout: A national survey in China. BMJ Open.

[29] Li H., Yuan B., Meng Q., Kawachi I. (2019). Contextual factors associated with burnout among Chinese primary care providers: A multilevel analysis. Int. J. Environ. Res. Public Health.

[30] Kan X. (1990). Village health workers in China: Reappraising the current situation. Health Policy Plan..

[31] Li T.T., Lei T., Sun F.N., Xie Z. (2017). Determinants of village doctors’ job satisfaction under China’s health sector reform: A cross-sectional mixed methods study. Int. J. Equity Health.

[32] Zhang S., Zhang W., Zhou H., Xu H., Qu Z., Guo M., Wang F., Zhong Y., Gu L., Liang X. (2015). How China’s new health reform influences village doctors’ income structure: Evidence from a qualitative study in six counties in China. Hum. Resour. Health.

[33] Thommasen H.V., Lavanchy M., Connelly I., Berkowitz J., Grzybowski S. (2001). Mental health, job satisfaction, and intention to relocate. Opinions of physicians in rural British Columbia. Can. Fam. Physician.

[34] Leigh R., Van Aarsen K., Foxcroft L., Lim R. (2020). P012: Does physician burnout differ between urban and rural emergency medicine physicians? A comparison using the Maslach Burnout Inventory tool. Can. J. Emerg. Med..

[35] Li C., Shi K., Luo Z., Li L., Yang Y. (2003). An investigation on job burnout of doctor and nurse. Chin. J. Clin. Psychol..

[36] Li H., Zuo M., Gelb A.W., Zhang B., Zhao X., Yao D., Xia D., Huang Y. (2018). Chinese anesthesiologists have high burnout and low job satisfaction: A cross-sectional survey. Anesth. Analg..

[37] Dyrbye L.N., West C.P., Shanafelt T.D. (2009). Defining Burnout as a Dichotomous Variable. J. General Internal Med..

[38] Brenninkmeijer V., VanYperen N. (2003). How to conduct research on burnout: Advantages and disadvantages of a unidimensional approach in burnout research. Occup. Environ. Med..

[39] O’Kelly F., Manecksha R.P., Quinlan D.M., Reid A., Joyce A., O’Flynn K., Speakman M., Thornhill J.A. (2016). Rates of self-reported ‘burnout’ and causative factors amongst urologists in Ireland and the UK: A comparative cross-sectional study. BJU Int..

[40] Maslach C., Jackson S.E., Leiter M.P. (2018). Maslach Burnout Inventory Manual.

[41] Shanafelt T.D., Oreskovich M.R., Dyrbye L.N., Satele D.V., Hanks J.B., Sloan J.A., Balch C.M. (2012). Avoiding burnout: The personal health habits and wellness practices of US surgeons. Ann. Surg..

[42] te Brake H., Bloemendal E., Hoogstraten J. (2003). Gender differences in burnout among Dutch dentists. Community Dent. Oral Epidemiol..

[43] Soler J.K., Yaman H., Esteva M., Dobbs F., Asenova R.S., Katić M., Ožvačić Z., Desgranges J.P., Moreau A., Lionis C. (2008). Burnout in European family doctors: The EGPRN study. Fam. Pract..

[44] Purvanova R.K., Muros J.P. (2010). Gender differences in burnout: A meta-analysis. J. Vocat. Behav..

[45] Siegrist J. (1996). Adverse health effects of high-effort/low-reward conditions. J. Occup. Health Psychol..

[46] Li J., Yang W., Cheng Y., Siegrist J., Cho S.-I. (2005). Effort—Reward imbalance at work and job dissatisfaction in Chinese healthcare workers: A validation study. Int. Arch. Occup. Environ. Health.

[47] Biaggi P., Peter S., Ulich E. (2013). Stressors, emotional exhaustion and aversion to patients in residents and chief residents—What can be done?. Swiss. Med. Wkly..

[48] Yang J., Zhang L. (2015). Relationship of job burnout and turnover intention of township hospital doctors in a county. China Med. Ethics.

[49] Wright J.G., Khetani N., Stephens D. (2011). Burnout among faculty physicians in an academic health science centre. Paediatr. Child Health.

[50] Duan X., Ni X., Shi L., Zhang L., Ye Y., Mu H., Li Z., Liu X., Fan L., Wang Y. (2019). The impact of workplace violence on job satisfaction, job burnout, and turnover intention: The mediating role of social support. Health Qual. Life Outcomes.

[51] Schaufeli Wilmar B., Leiter Michael P., Maslach C. (2009). Burnout: 35 years of research and practice. Career Dev. Int..

